# Extended secondhand smoke exposure alters potassium channel activity and reduces excitability of cardiac vagal neurons in mice

**DOI:** 10.3389/fphys.2026.1907874

**Published:** 2026-08-19

**Authors:** Junqing Sun, Shiyue Pan, Emma Karey, Kent E. Pinkerton, Chao-Yin Chen

**Affiliations:** 1 Department of Pharmacology, University of California, Davis, Davis, CA, United States; 2 Center for Health and the Environment, University of California, Davis, Davis, CA, United States

**Keywords:** autonomic function, cardiovascular, environmental tobacco smoke, neuroplasticity, nucleus ambiguus

## Abstract

**Background:**

Despite the decline in secondhand smoke (SHS) exposure since the implementation of smoking bans in public places, SHS exposure remains a significant health risk factor affecting about 1/3 of non-smokers worldwide. We previously showed that 12 weeks of SHS exposure reduces heart rate variability, an effect that peaks at week 4. We further showed that 4 weeks of SHS exposure significantly reduces cardiac vagal neuron’s (CVN) excitability that is associated with a reduced small conductance calcium-dependent potassium (SK) channel activity.

**Objectives:**

This study aimed to test whether the reduced excitability in CVNs also wanes with longer exposure duration (12 weeks) and whether 4-aminopyridine sensitive voltage-gated potassium channels contribute to the SHS-induced decreases in neuronal excitability.

**Methods:**

Adult male mice were exposed to 12 weeks of filtered air or SHS at an environmental-relevant concentration (3 mg/m^3^, 6 hr/d, 5d/wk). We performed whole-cell patch-clamp recordings on anatomically identified CVNs in the nucleus ambiguus.

**Results:**

12 weeks of SHS exposure significantly increased action potential (AP) thresholds and reduced spiking responses to excitation. SHS exposure did not significantly alter resting membrane potential, or 4-aminopyridine sensitive channel activity, suggesting that leak potassium channels and voltage gated potassium channels were unlikely to contribute to the reduced excitability. We found two adaptations that may serve to counteract the reduced excitability. First, APs inactivated at higher voltages that helped to maintain the spiking response range and increase maximum discharge frequency. Second, blocking SK channels with apamin had smaller effects on the spiking response in CVNs from SHS-exposed mice, suggesting that a reduced SK channel activation during spiking activity may help to dampen the reduced excitability.

**Conclusion:**

Environmentally relevant SHS exposure reduces neuronal excitability of CVNs through mechanisms other than enhanced SK and voltage-gated potassium channel functions.

## 1 Introduction

Secondhand smoke (SHS) exposure has declined since 1990 largely due to smoking bans in public places and increased awareness of the harmful effects of SHS (Kelleher and Frazer, 2014; Meyers et al., 2009). However, this downward trend plateaued in 2016 (Liu et al., 2025). About 30-39% of non-smokers were exposed to SHS worldwide in 2021, although exposure levels vary greatly across countries and different sociodemographic (Su et al., 2025). Cardiovascular causes (e.g. sudden cardiac death, atrial fibrillation, ischemic heart disease and stroke) are the leading causes of death attributable to SHS exposure (Leung et al., 2024; Lin et al., 2022; Max et al., 2012; Oberg et al., 2011).

Epidemiological studies show that the most detrimental SHS exposure-related cardiovascular morbidity and mortality is sudden cardiac death (Glantz and Parmley, 1995; Thun et al., 1999). SHS-induced decreases in autonomic regulation (which manifests as a decreased heart rate variability [HRV]) is a significant risk factor; it’s arrhythmogenicity is a major cause of sudden cardiac death (Zipes and Wellens, 1998). It is well documented that both acute and chronic SHS exposure can decrease HRV (Barnoya and Glantz, 2005; Chen et al., 2008; Pope et al., 2001; Wilson et al., 2010; Zhang et al., 2013). Previous work in our laboratory confirmed that environmentally relevant SHS exposure (3 mg/m^3^ of total suspended particles [TSP]) significantly reduced short-term HRV (rMSSD, root mean square of successive difference), a measure of cardiac vagal regulation, in a mouse model. The SHS-induced decrease in short-term HRV is greatest in week 4 of the 12-week exposure (-32% vs. -16%, week 4 vs. week 12, respectively) (Pan et al., 2023).

In the central nervous system, the cell bodies of cardiac vagal neuron (CVN) are largely located in the nucleus ambiguus (NA) in the ventrolateral medulla (Mendelowitz, 1999). These neurons are not pacemaker cells, so their neuronal output is shaped by their intrinsic excitability integrating synaptic inputs (Mendelowitz, 1996). We previously showed that four weeks of SHS exposure reduces the intrinsic excitability of anatomically identified CVNs in the NA, suggesting that the SHS-induced reduction in excitability of CVNs may underlie the decrease in HRV following SHS exposure (Sun et al., 2021). However, we do not know whether these changes in CVNs persist with longer exposure duration (12 weeks) when the magnitude of the SHS-induced decrease in short-term HRV wanes.

Both small conductance calcium-dependent potassium (SK) and 4-aminopyridine sensitive voltage-gated transient potassium currents have been shown to modulate CVN’s intrinsic excitability, shape the action potential, and determine the spike discharge pattern (Mendelowitz, 1996; Mihalevich et al., 1996). SK channels are activated by elevated intracellular calcium during an action potential, resulting in membrane hyperpolarization that limits the firing frequency (Bond et al., 1999, 2005). 4-aminopyridine sensitive voltage-gated potassium (A-type) channels are fast activating and inactivating channels. They are activated at subthreshold voltage (membrane voltage more negative than the action potential threshold). Activation of the A-type channels increases action potential threshold, fall rate, and afterhyperpolarization. As a result, activation of A-type channels decreases action potential halfwidth and neuronal discharge frequency.

We previously showed an unexpected finding that the reduced CVN’s excitability after 4 weeks of SHS exposure was accompanied by compensatory attenuation, not enhancement, of SK channel activity, suggesting that other mechanisms contributing the reduced excitability (Sun et al., 2021). Interestingly, the SHS-induced decrease in intrinsic excitability was associated with a right-ward shift in action potential (AP) inactivation voltage (Sun et al., 2021). However, it was unclear whether voltage-gated potassium channels contribute to the reduced intrinsic excitability and/or right-ward shift in AP inactivation voltage. This study aimed to test whether 1) the reduced excitability in CVNs wanes with longer SHS exposure duration (12 weeks) and 2) blocking 4-aminopyridine sensitive voltage-gated potassium currents eliminated the SHS exposure-induced decrease in neuronal excitability.

## 2 Materials and methods

All animal related work and procedures were approved by the University of California Davis Institutional Animal Care and Use Committee and in compliance with the Animal Welfare Act and Public Health Service Policy on Humane Care and Use of Laboratory Animals. All animals were treated humanely that is consistent with the US National Institutes of Health agency’s guidelines. Mice were provided with free access to regular rodent chow and water in a 12-hour light/12-hour dark cycle environment. The relative humidity of the animal housing room was 63 ± 20% (mean ± SD) with average room temperature of 21 ± 1 °C (mean ± SD).

Under isoflurane anesthesia (1.5-5% in 100% oxygen), male C57BL/6J mice (7–8 weeks old) underwent surgeries for retrograde labeling of CVNs with a fluorescent dye, DiI (1, 1’-dioctadecyl 3, 3, 3’, 3’-tetramethylindo-carbocyanine perchlorate). As previously described, a parafilm patch (1 mm^3^) coated with DiI was placed over the sinoatrial node region through a right thoracotomy (Pham et al., 2009; Sun et al., 2021). The parafilm patch was secured and sealed with tissue glue and the chest was closed. The animals were given buprenorphine (0.05 mg/kg) preemptively and every 12 hours for two post-op days for pain control.

### 2.1 SHS exposure

Mice were randomly assigned to the filtered air- (FA) or SHS-exposed group two weeks after the surgery. As in previous studies, exposure was conducted 6 hours/day, 5 days/week for 12 weeks (Bonham et al., 2001; Chen et al., 2008; Sekizawa et al., 2008; Sun et al., 2021; Wang et al., 2018). Conditioned 3R4F research cigarettes were obtained from the University of Kentucky Tobacco and Health Research Institute (Lexington, KY, USA). Cigarette smoke was generated with a modified ADL/II system (Little Cambridge, MA, USA) under Federal Trade Commission conditions (35 cm^3^ puff over 2 seconds every minute). The target concentration (3 mg/m^3^ of TSP) was achieved by mixing the generated smoke with FA in a mixing and aging chamber prior to delivery to the exposure chamber. Whole body exposure was performed in a 0.44 m^3^ glass Hinners-type exposure chamber with mice in their home cage with wire lids, rodent chow and water *ad libitum*. Major components of cigarette smoke were characterized daily and were (mean ± SD): TSP (3.0 ± 0.1 mg/m^3^); Nicotine (0.4 ± 0.2 mg/m^3^); and carbon monoxide (14 ± 1 ppm).

### 2.2 Whole cell patch clamp recordings

The day after exposures ceased, mice were anesthetized with 5% isoflurane and decapitated for brain slices. Brainstem was sliced (150 μm, transverse) using a vibrating microtome (Leica microsystems, Inc. Bannockburn, IL) in ice-cold high sucrose artificial cerebrospinal fluid (aCSF) that contained (in mM): 73 NaCl, 2.5 KCl, 2 MgCl_2_, 1.25 NaH_2_PO4, 25 NaHCO_3_, 24 dextrose, 0.5 CaCl_2_ and 75 sucrose (300 mOSM, continuously bubbled with 95%O_2_–5%CO_2_). Brain slices were incubated in high sucrose aCSF for 45 minutes at 35 °C before transferring to regular aCSF at room temperature that contained (mM) 128 NaCl, 2.5 KCl, 1 MgCl_2_, 1.25 NaH_2_PO4, 25 NaHCO_3_, 10 dextrose and 2 CaCl_2_ (300 mOSM, continuously bubbled with 95%O_2_–5%CO_2_).

**Figure 1:**
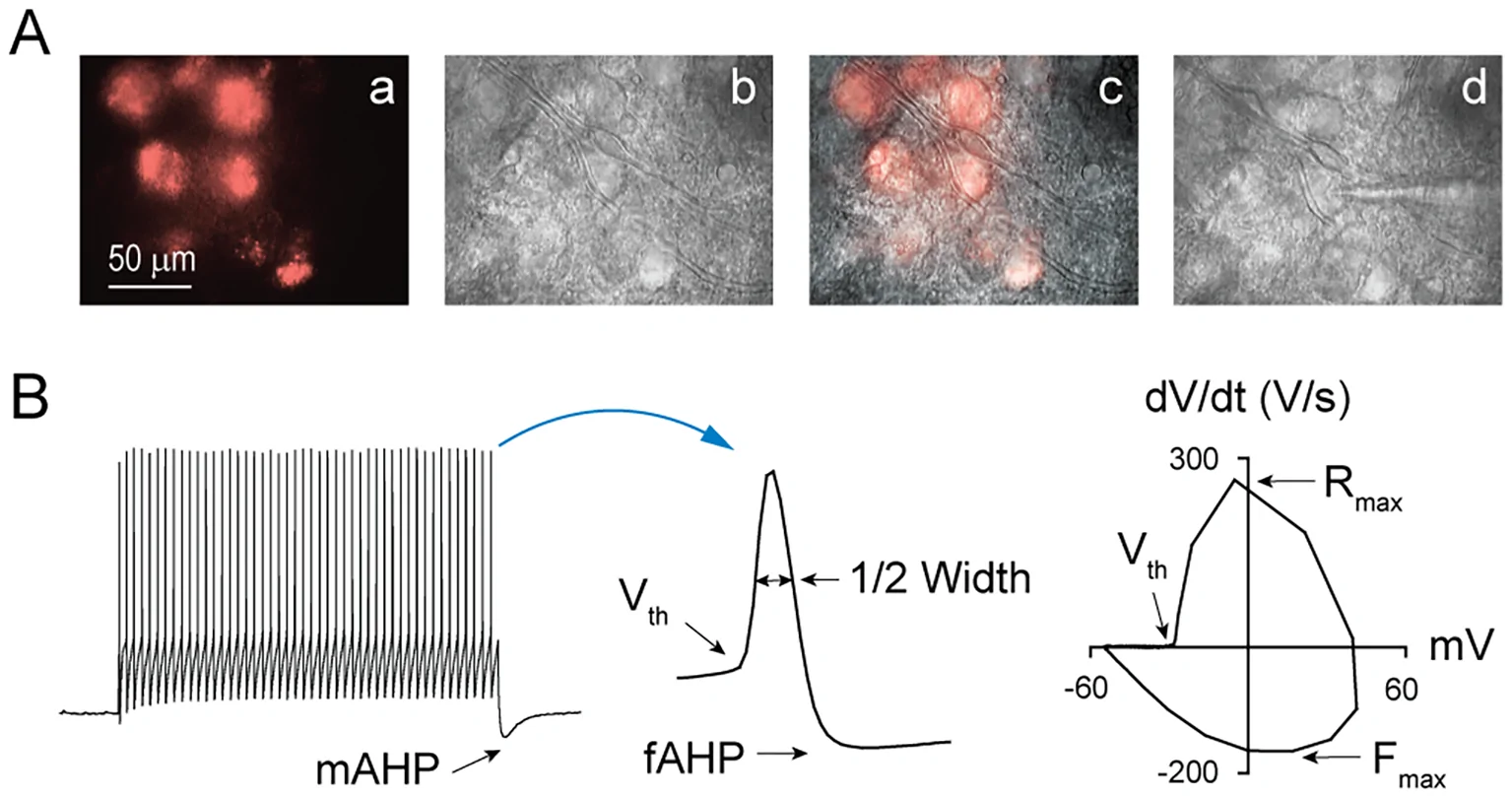
Example images of cardiac vagal neurons (CVNs) with retrograde labeling and action potential characteristic measurements. **(A)** Nucleus ambiguous region viewed at 40x with a fluorescence filter for DiI (a), infrared-differential interference contrast (IR-DIC) (b), overlay of the IR-DIC and fluorescence images (c), and an identified neuron with a patch electrode in whole-cell configuration (d). **(B)** An example trace of action potentials evoked by a 1-s depolarizing current step (left) demonstrates the medium afterhyperpolarization (mAHP). The action potential waveform (middle panel) and its phase plane plot (right panel) illustrate the action potential characteristic measurements: voltage threshold (V_th_), fast afterhyperpolarization (fAHP), maximum rise rate (R_max_), and maximum fall rate (F_max_).

Whole cell patch recordings were performed in regular aCSF at 33–34 °C on fluorescently labeled CVNs in the NA (**Figure 1A**), identified with an optical filter set for DiI (XF 108, Omega Optical Inc., Brattleboro, VT). After giga ohm seal, current clamp recordings were made with pipette solution contained (in mM): 10 KCl, 128 K-gluconate, 2 MgCl_2_, 10 HEPES, 5.5 EGTA, 2 Na_2_-ATP and 0.1 CaCl_2_. Signals were recorded with a MultiClamp 700B amplifier (Axon instruments, Sunnyvale, CA), filtered at 2 kHz, and digitized at 10 kHz with a Digidata 1440A (Axon Instruments).

Resting membrane potential was measured immediately after whole-cell configuration and the neuron was current-clamped at -60 mV. One-second depolarizing current steps (100–700 pA in 100 pA increments) were used to determine spiking adaptation characteristics and post-train medium afterhyperpolarization (mAHP, **Figure 1B**). A five-second depolarizing current ramp (1 nA/s) was used to determine the voltage/current threshold for generating an AP and AP characteristics at membrane voltages that induce inactivation of voltage-gated ion channels. Measured AP characteristics (**Figure 1B**) included: AP amplitude, intra-train fast AHP (fAHP) amplitude, maximum AP rise rate, maximum AP fall rate, and AP ½ width. In some neurons, the protocols were repeated with a selective SK channel blocker (20 nM apamin) or a non-selective voltage-gated potassium channel blocker (1 mM 4-aminopyridine).

### 2.3 Data analysis

Data are expressed as mean ± SE and analyzed with GraphPad Prism (GraphPad Software, Inc.) In the absence of blockers, a *t*-test was used to compare current threshold, voltage threshold, resting membrane potential, maximum spiking frequency, maximum AP rise rate, and maximum AP fall rate between FA and SHS groups. Spiking responses and AP characteristics were analyzed with a 2-way repeated ANOVA or a Mixed-effects analysis (as specified in the results section). Data presented in tables were analyzed with a 2-way repeated ANOVA. Significance was set at *p* < 0.05.

## 3 Results

### 3.1 Spiking response to step current injections

**Figure 2:**
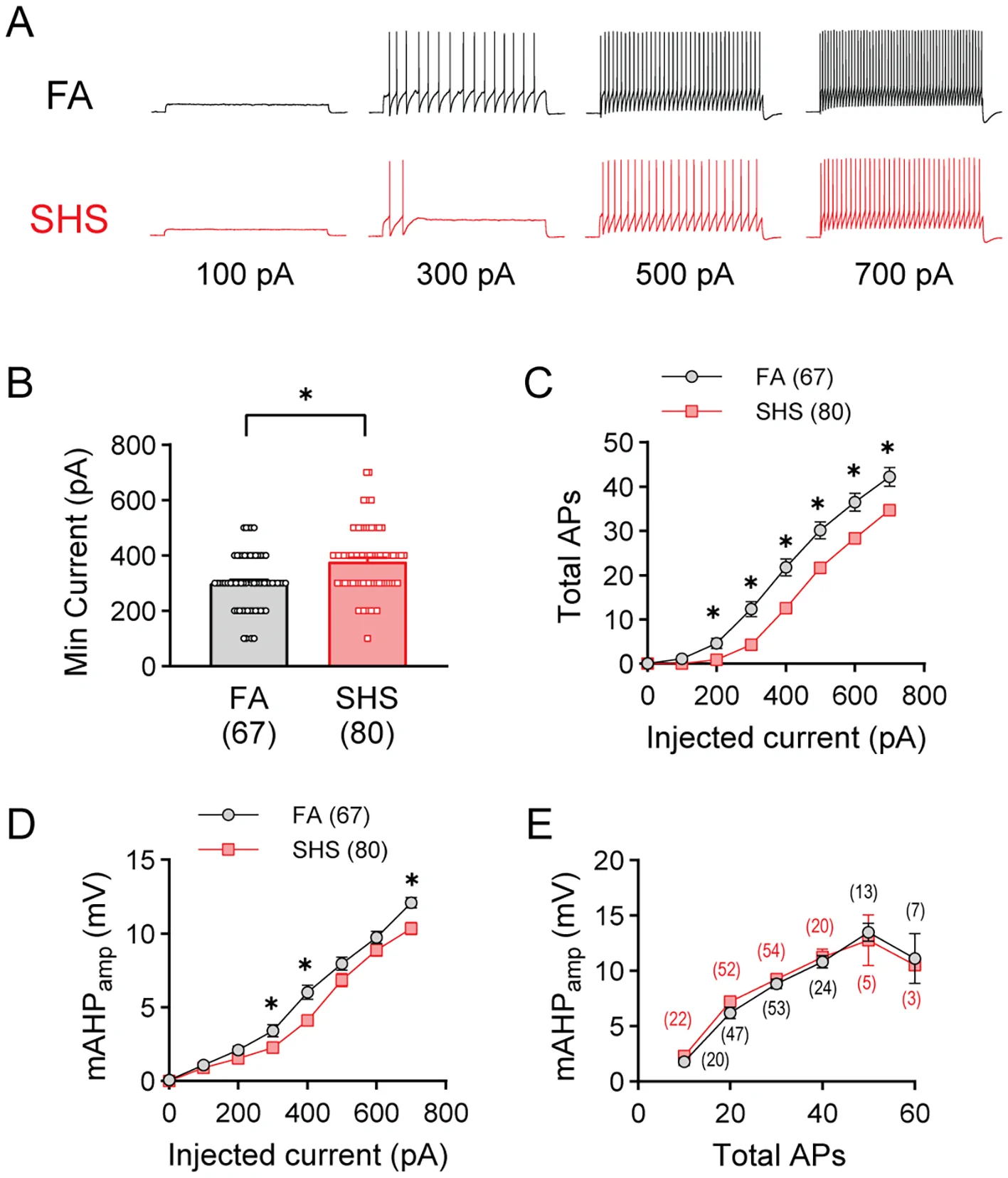
Neuronal response to 1-s depolarizing current steps from filtered air (FA) and secondhand smoke (SHS) mice. **(A)** Example traces of action potentials evoked by 1-s depolarizing current steps. **(B)** The minimum current step required to evoke an action potential was significantly higher in CVNs from the SHS group (*t*-test, *p* < 0.05). **(C)** Total number of action potentials (total APs) discharged to increasing depolarizing current steps showing a reduced spiking response in CVNs from the SHS group, compared to those from the FA group. **(D)** mAHP amplitudes at the same depolarizing current step were significantly smaller in CVNs from the SHS group, compared to those from the FA group. **(E)** mAHP amplitudes with the same total number of action potentials were not different between two exposure groups. Numbers in parentheses indicate number of neurons. Full ANOVA p-values are in **Supplementary Table 1**. ^*^p < 0.05, FA vs. SHS, Fisher’s LSD test.

All experiments were performed on fluorescently (DiI) labeled CVNs in the NA (**Figure 1A**); and no significant difference in resting membrane potential was observed between exposure groups (**Supplementary Figure 1A**). Examples of neuronal spiking responses to increasing injected current steps from both exposure groups are shown in **Figure 2A**. The lowest current step that triggered AP generation was significantly higher (*p* < 0.05, *t*-test) in CVNs from the SHS group, compared to those from the FA group (**Figure 2B**). CVNs from the SHS group consistently discharged fewer APs than those from the FA group (**Figure 2C**). A higher degree of spike adaptation is unlikely to explain the reduced spiking response observed in SHS neurons as there was no significant difference in the frequency adaptation pattern between the two groups (**Supplementary Figures 1B, C**). The reduced spiking response in SHS neurons was also associated with a smaller mAHP amplitude at the same injected current (**Figure 2D**). However, mAHP amplitudes did not differ significantly between exposure groups at the same total discharged AP numbers (**Figure 2E**), suggesting that mAHP amplitude and spiking frequency are tightly coupled.

### 3.2 Spiking response to ramp current injections

**Figure 3:**
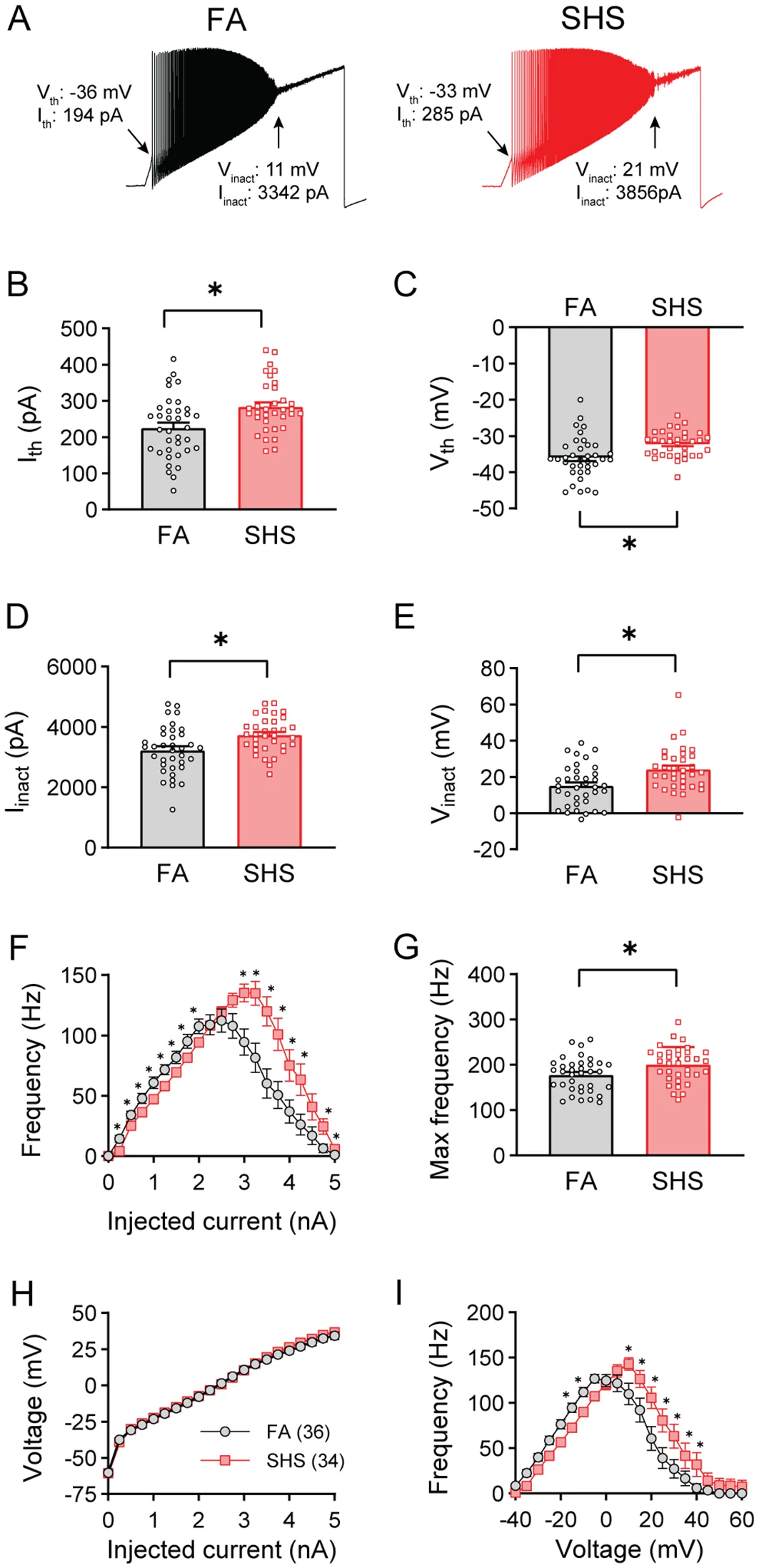
Neuronal response to a ramp current injection (5 nA over 5 s) from filtered air (FA, n=36)- and secondhand smoke (SHS, n=34)-exposed mice. **(A)** Example traces of action potentials evoked by a 5-s depolarizing current ramp to 5 nA. Arrows point to the voltage/current threshold where the first action potential was discharged and AP inactivation voltage/current. **(B)** Current threshold (I_th_) for action potential generation was significantly higher in CVNs from SHS-exposed mice (*t*-test, *p* < 0.05). **(C)** Voltage threshold (V_th_) was also significantly higher in the SHS-exposed group (*t*-test, *p* < 0.05). **(D)** Inactivation current (I_inact_) was significantly higher in the SHS group (*t*-test, *p* < 0.05). **(E)** Inactivation voltage (V_inact_) was significantly higher in the SHS group (*t*-test, *p* < 0.05). **(F)** Discharge frequency during the 5-s depolarizing current ramp showing a right-ward shift in the firing frequency to increasing current injection in the SHS-exposed group. **(G)** Maximum instantaneous frequency was higher in CVNs from the SHS-exposed group (*t*-test, *p* < 0.05). **(H)** Membrane voltage during the 5-s depolarizing ramp current injection showing no exposure effect in membrane voltage response to injected current. **(I)** Discharge frequency at different membrane voltages during the 5-s depolarizing current ramp showing a right-ward shift in the firing frequency to higher membrane voltages in the SHS-exposed group (n=26–36 for FA, n=17–34 for SHS). Numbers in parentheses indicate number of neurons. Full ANOVA p-values are in **Supplementary Table 2**. ^*^p < 0.05 FA vs. SHS, Fisher’s LSD test.

Examples of neuronal spiking responses to a ramp current injection from both groups are shown in **Figure 3A**. The instantaneous frequency increased progressively as the ramp current increased, until AP inactivated at higher current levels (**Figure 3A**). CVNs from the SHS group had significantly higher current (**Figure 3B**) and voltage (**Figure 3C**) thresholds, compared to FA CVNs (*p* < 0.05, *t*-test). AP inactivation occurred at higher current (**Figure 3D**) and membrane voltage (**Figure 3E**) in the SHS group (*p* < 0.05, *t*-test). At the spiking frequency rising phase, CVNs from the SHS group had a flatter input-output relationship (**Figure 3F**), consistent with the observation from the step current injection protocol (**Figure 2C**). However, CVNs from the SHS group reached the inactivation phase at higher injected current, shifting the curve to the right (**Figure 3F**), with a significantly higher maximum discharge frequency (**Figure 3G**). This shift to higher injected current was not due to differences in membrane voltage at the same injected current because there was no significant difference in the membrane voltage–current relationships between the two groups (**Figure 3H**). Plotting the instantaneous frequency against membrane voltage confirmed a shift in the frequency-voltage relationship curve to higher membrane voltage in CVNs from the SHS group (**Figure 3I**).

**Figure 4:**
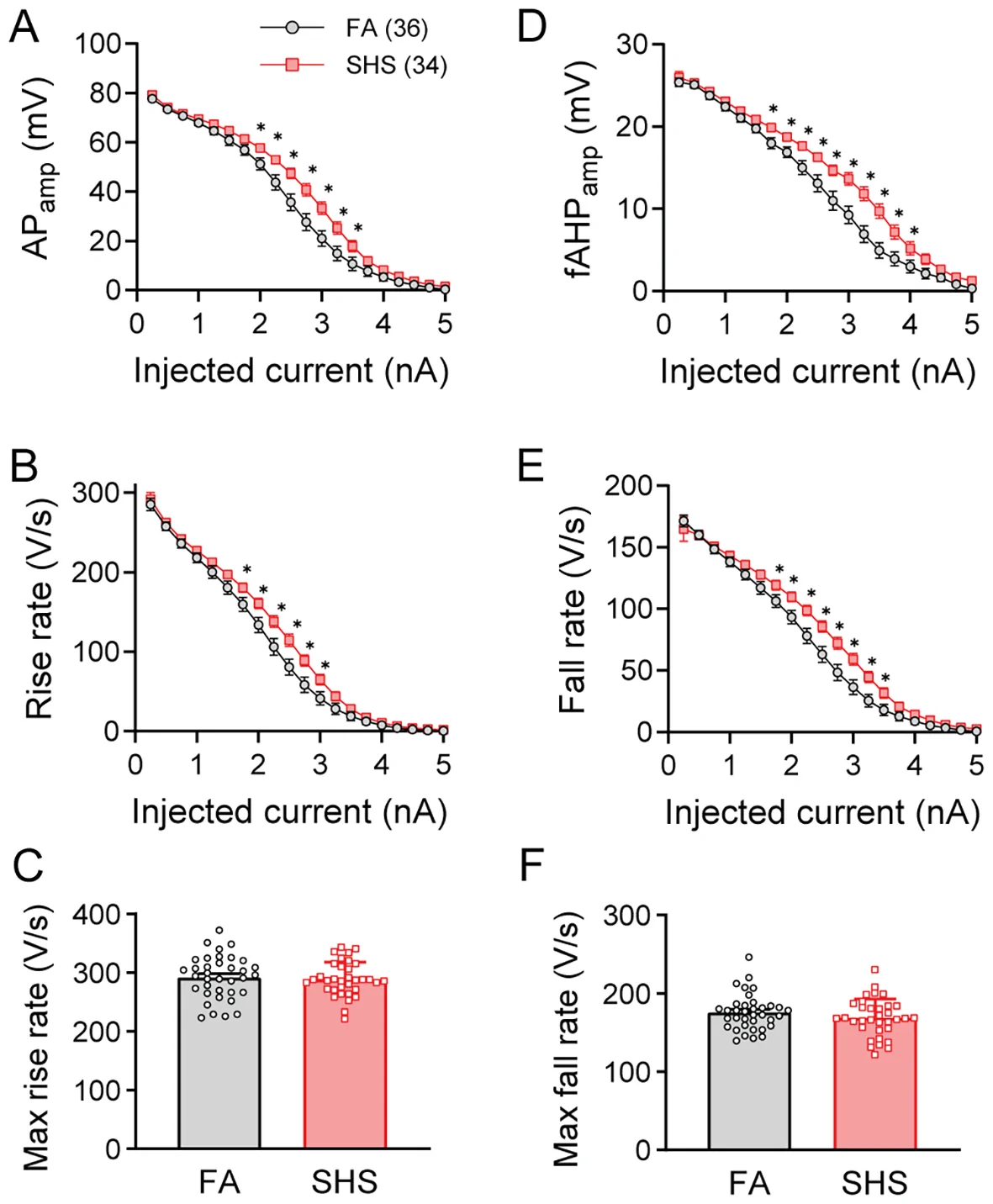
Action potential (AP) characteristics from FA (n=36) and SHS (n=34) exposed mice. **(A)** AP amplitude decreased as injected current increased. There was a right-ward shift in the AP amplitude decay curve in the SHS group, resulting in larger amplitudes at higher injected currents. **(B)** AP rise rate curve showed a similar right-ward shift in the SHS group, resulting in faster rise rates at higher injected currents. **(C)** There was no difference in maximum rise rate between the two groups (*t*-test, *p* > 0.05). **(D)** fAHP amplitudes were larger at higher injected currents in the SHS group. **(E)** AP fall rates were faster in the SHS group. **(F)** There was no difference in the maximum fall rate between the two groups (*t*-test, *p* > 0.05). Numbers in parentheses indicate number of neurons. Full ANOVA p-values are in **Supplementary Table 3**. ^*^p < 0.05 FA vs. SHS, Fisher’s LSD test.

The right-ward shift in the frequency–current relationship in the SHS group (**Figure 3F**) was accompanied by a right-ward shift in the AP amplitude–current relationship (**Figure 4A**) and AP rise rate–current relationship (**Figure 4B**) with no change in the maximum rise rate (**Figure 4C**). Similarly, the fAHP amplitude (**Figure 4D**) and AP fall rate (**Figure 4E**) curves also shifted to the right with no significant difference in the maximum fall rate between the two groups (**Figure 4F**). Plotting these AP parameters against membrane voltage confirmed that AP amplitude, rise rate, fAHP, and fall rate curves shifted to higher membrane voltage in CVNs from the SHS group (**Supplementary Figure 2**). Consequently, CVNs from the SHS group had more compact APs (narrower ½ width) at higher injected currents and membrane voltages (**Supplementary Figure 2**).

### 3.3 Potassium channel blockers on spiking response to step current injections

**Figure 5:**
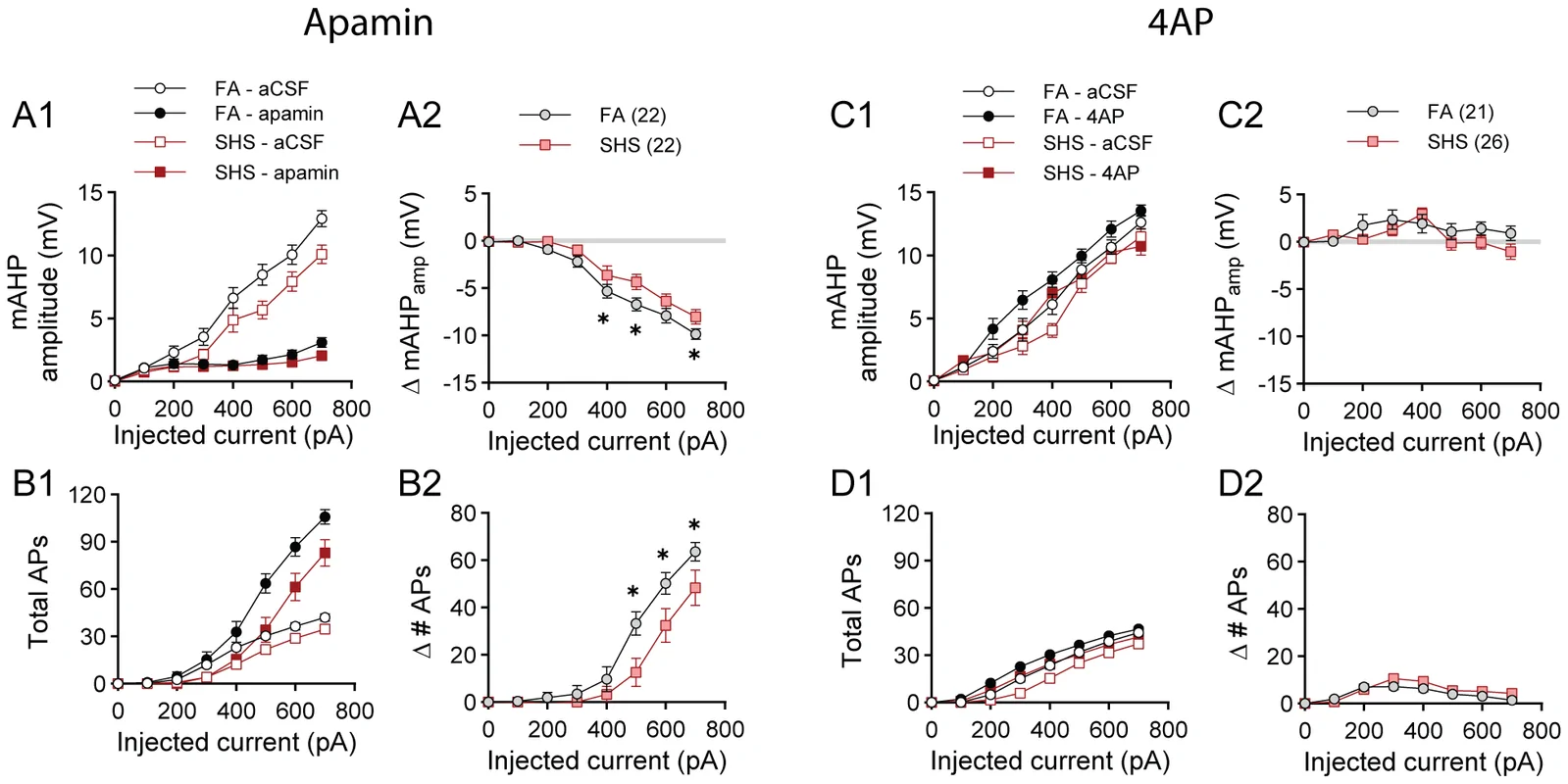
Effects of apamin and 4-aminopyridine (4AP) on mAHP and spiking responses to step current injections. **(A1)** Apamin significantly reduced mAHP amplitude in both exposure groups. **(A2)** Apamin-induced changes in mAHP amplitude (apamin – aCSF) is significantly smaller in the SHS group. **(B1)** Apamin significantly increased Total number of APs in both groups. **(B2)** Apamin-induced changes in total number of APs (apamin – aCSF) is significantly smaller in the SHS group. **(C1)** 4AP increased mAHP amplitude in both exposure groups. **(C2)** 4AP-induced changes in mAHP amplitude (4AP – aCSF) were not different between the two exposure groups. **(D1)** 4AP increased total number of APs in both groups. **(D2)** 4AP-induced changes in total number of APs (4AP – aCSF) were not different between the two exposure groups. Numbers in parentheses indicate number of neurons. Full ANOVA p-values are in **Supplementary Tables 4** and **5**. ^*^p < 0.05 FA vs. SHS, Fisher’s LSD test.

Application of apamin significantly increased the minimum current step to evoke APs (**Table 1**). There was a trend (*p* = 0.08, *t*-test) for a greater apamin-induced increase in current threshold in the SHS group (+41 ± 18 pA vs. +77 ± 17 pA, FA vs. SHS, respectively). As expected, blocking SK channels with apamin eliminated the mAHP (**Figure 5A1**) in CVNs from both FA and SHS groups, confirming that mAHP is mediated by activation of SK channels. Apamin-induced decreases in mAHP amplitude (**Figure 5A2**) were significantly smaller in the SHS group. Application of apamin significantly increased spiking response to step current injections in both exposure groups (**Figure 5B1**). In the presence of apamin, the SHS group still showed a lower spiking response compared to the FA group, suggesting mechanism(s) beyond SK channels contributed to the reduced neuronal excitability observed in SHS CVNs. The apamin-induced increases in spiking response were significantly smaller in the SHS group (**Figure 5B2**).

**Table 1:** Effects of apamin and 4-aminopyridine (4AP) on current and voltage thresholds.

Measurement	FA			SHS			ANOVA *p*-values		
	aCSF	Blocker	n	aCSF	Blocker	n	Exposure	Blocker	Interaction
Step protocol									
I_th_ (apamin)	277 ± 15	318 ± 25	22	382 ± 21	459 ± 27	22	0.0001	<0.0001	0.1554
I_th_ (4AP)	271 ± 18	167 ± 14	21	342 ± 20	212 ± 21	26	0.0186	<0.0001	0.3430
Ramp protocol: apamin									
I_th_ (pA)	209 ± 19	282 ± 27	12	304 ± 24	427 ± 33	10	0.0027	<0.0001	0.0335
I_inact_ (nA)	3.4 ± 0.2	2.3 ± 0.2	12	3.8 ± 0.2	3.0 ± 0.3	10	0.0931	<0.0001	0.4571
V_th_ (mV)	-37 ± 1	-32 ± 1	12	-32 ± 1	-26 ± 2	10	0.0037	<0.0001	0.2901
V_inact_ (mV)	13 ± 4	11 ± 3	12	23 ± 4	30 ± 5	10	0.0057	0.2505	0.0919
Ramp protocol: 4AP									
I_th_ (pA)	198 ± 20	166 ± 11	10	267 ± 21	213 ± 16	11	0.0192	0.0004	0.2779
I_inact_ (nA)	3.1 ± 0.2	2.4 ± 0.2	10	3.8 ± 0.2	3.1 ± 0.2	11	0.0121	<0.0001	0.6673
V_th_ (mV)	-38 ± 1	-39 ± 2	10	-33 ± 1	-35 ± 1	11	0.0073	0.0407	0.6461
V_inact_ (mV)	15 ± 3	18 ± 4	10	22 ± 3	31 ± 4	11	0.0241	0.0216	0.2635

I_th_, current threshold; V_th_, voltage threshold; I_inact_, inactivation current; V_inact_, inactivation voltage.

Application of 4-aminopyridine significantly decreased the minimum current step required to evoke APs (**Table 1**). There was no significant difference (*p* > 0.05, *t*-test) in 4-aminopyridine-induced reduction in current threshold between the two exposure groups (-105 ± 15 pA vs. -131 ± 21 pA, FA vs. SHS, respectively). In contrast to the effects of apamin, 4-aminopyridine increased mAHP amplitude (**Figure 5C1**), likely due to a small increase in spiking response to current injections (**Figure 5D1**). There was no significant difference in 4-aminopyridine-induced increase in mAHP amplitude and spiking response between FA and SHS groups (**Figures 5C2, D2**).

### 3.4 Potassium channel blockers on spiking response to ramp current injections

Similar to the results from step current injections, apamin significantly increased current and voltage thresholds determined with the ramp protocol (**Table 1**). The apamin-induced increase in current threshold was significantly greater (*p* < 0.05, *t*-test) in the SHS group (+73 ± 16 pA vs. +124 ± 15 pA, FA vs. SHS, respectively) while SHS exposure did not (*p* > 0.05, *t*-test) affect apamin-induced increases in voltage threshold (+4.7 ± 1.3 mV vs. +6.5 ± 0.9 mV, FA vs. SHS, respectively). Apamin significantly decreased inactivation current similarly in both exposure groups while having no consistent effects on inactivation voltage (**Table 1**).

**Figure 6:**
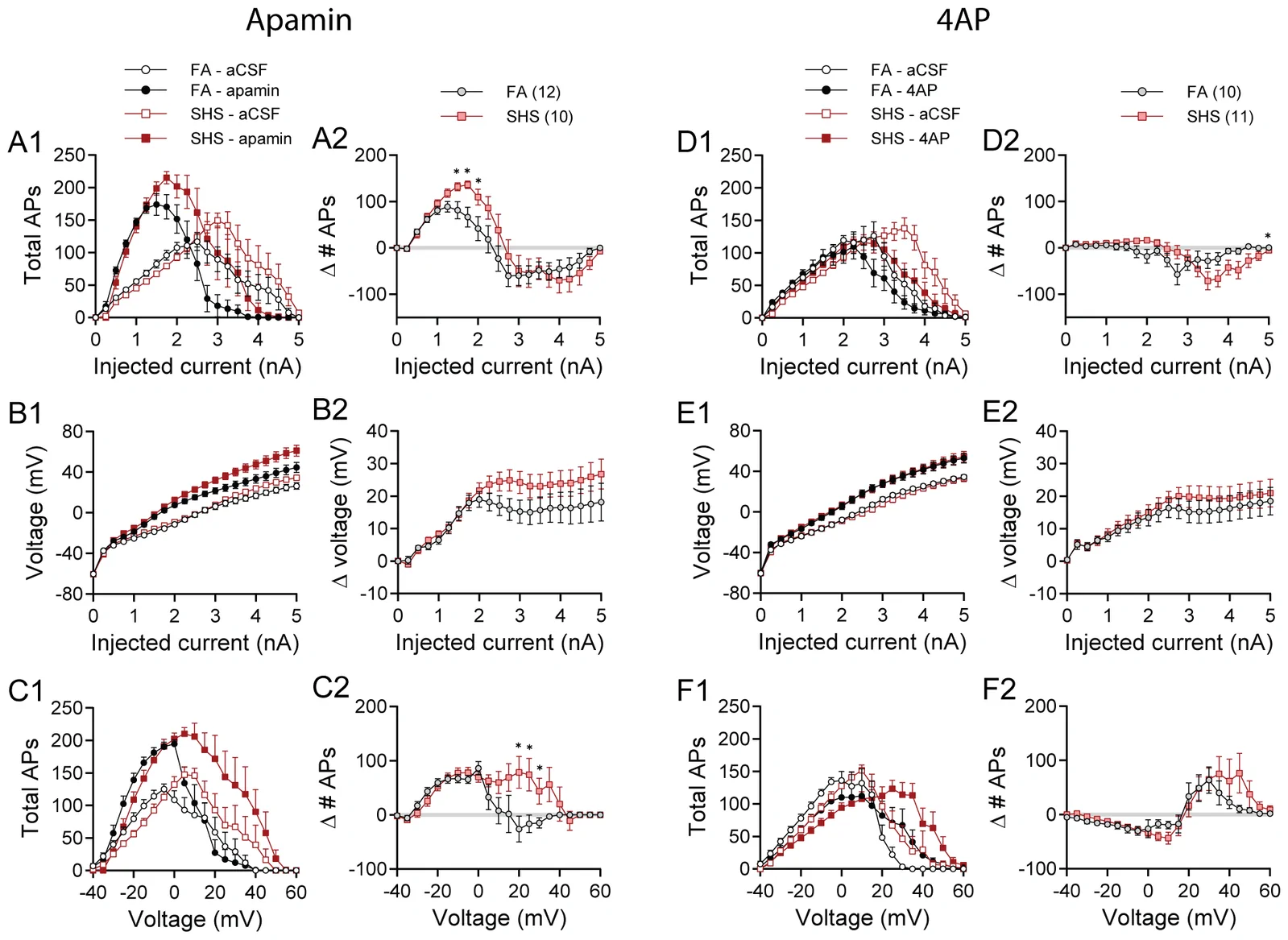
Effects of apamin and 4-aminopyridine (4AP) on spiking responses to ramp current injections. **(A1)** CVNs discharged more action potentials in the presence of apamin at injected currents below ~2 nA, followed by lower discharge rates at injected currents above ~2 nA. **(A2)** The apamin-induced changes in spiking activity (apamin – aCSF) curve shifted to the right in the CVNs from the SHS group. **(B1)** Membrane voltages at the same injected currents were higher in the presence of apamin in both exposure groups. **(B2)** There was no difference between the two exposure groups in the apamin-induced changes in membrane voltage (apamin – aCSF). **(C1)** Apamin increased neuronal discharge rate in both exposure groups. **(C2)** Apamin-induced increases in spiking activity persisted at higher membrane voltages in the SHS group. **(D1)** 4AP decreased the discharge rates at higher injected currents. **(D2)** The 4AP-induced changes in spiking activity (4AP – aCSF) curve shifted to the right in the SHS group. **(E1)** Membrane voltages at the same injected currents were higher in the presence of 4AP for both exposure groups. **(E2)** There was no difference between the two exposure groups in 4AP-induced changes in membrane voltage (4AP – aCSF). **(F1)** Effects of 4AP on neuronal discharge rate at increasing membrane voltages. **(F2)** There was no significant difference between the two exposure groups in 4AP-induced changes in spiking response (4AP – aCSF). Numbers in parentheses indicate number of neurons. Full ANOVA p-values are in **Supplementary Tables 6** and **7**. ^*^p < 0.05 FA vs. SHS, Fisher’s LSD test.

For spiking response, apamin significantly increased the spiking activity in the frequency rising phase and lowered the current at which the CVNs entered the inactivation phase (**Figure 6A1**). As a result, the effect of apamin on spiking frequency over the entire ramp showed a biphasic pattern – a higher spiking frequency in the rising phase followed by a lower spiking frequency in the inactivation phase (**Figure 6A2**). Apamin significantly increased the maximum spiking frequency (**Table 2**); this increase was not significantly different (*p* > 0.05, *t*-test) between the exposure groups (58 ± 7 Hz vs. 71 ± 7 Hz, FA vs. SHS, respectively).

**Table 2:** Effects of apamin and 4-aminopyridine (4AP) on maximum frequency (Hz), rise rate (V/s), and fall rate (V/s).

Measurement	FA			SHS			ANOVA *p*-values		
	aCSF	Blocker	n	aCSF	Blocker	n	Exposure	Blocker	Interaction
Apamin									
Frequency	168 ± 10	226 ± 9	12	203 ± 16	273 ± 14	10	0.0205	<0.0001	0.2248
Rise rate	308 ± 9	281 ± 10	12	288 ± 8	257 ± 9	10	0.0749	<0.0001	0.6978
Fall rate	183 ± 8	177 ± 6	12	177 ± 8	170 ± 6	10	0.5184	0.0516	0.9079
4AP									
Frequency	184 ± 11	231 ± 17	10	203 ± 7	219 ± 8	11	0.4959	0.0001	0.0245
Rise rate	293 ± 8	264 ± 15	10	300 ± 10	268 ± 15	11	0.7201	0.0018	0.9325
Fall rate	178 ± 4	131 ± 8	10	162 ± 7	127 ± 5	11	0.2200	<0.0001	0.2468

The shift in the inactivation phase to a lower injected current in the presence of apamin was due to a steeper membrane voltage response to injected current during apamin perfusion (**Figure 6B1**). There was no significant difference in the apamin-induced steeper membrane voltage response between the two groups (**Figure 6B2**). Plotting instantaneous frequency against membrane voltage confirmed that the increased spiking activity persisted at much higher membrane voltages in CVNs from the SHS group (**Figure 6C**).

Application of 4-aminopyridine significantly reduced current and voltage thresholds, as well as inactivation current but with significantly higher inactivation voltage (**Table 1**). There was no significant difference (*p* > 0.05, *t*-test) between the two groups in the 4-aminopyridine-induced decrease in current threshold (-107 ± 14 pA vs. -152 ± 15 pA, FA vs. SHS, respectively), voltage threshold (-1.3 ± 1.4 mV vs. -2.1 ± 0.7 mV, FA vs. SHS, respectively), inactivation current (-642 ± 75 pA vs. -692 ± 85 pA, FA vs. SHS, respectively), or increase in inactivation voltage (3.3 ± 3.4 mV vs. 9.0 ± 3.5 mV, FA vs. SHS, respectively).

4-aminopyridine did not have significant effect on neuronal discharge rate in the frequency rising phase, but the neurons reached the inactivation phase at lower injected currents, as indicated by a decrease in spiking activity at higher injected currents (**Figure 6D**). The membrane voltage response to injected current was steeper in the presence of 4-aminopyridine (**Figure 6E1**), however, there was no difference between the FA and SHS groups (**Figure 6E2**). Plotting spiking repose against membrane voltage revealed that 4-aminopyridine induced an initial reduction in spiking activity at lower voltages, followed by a higher spiking activity at higher voltages (**Figure 6F**). 4-aminopyridine significantly increased the maximum spiking frequency (**Table 2**), an increase that was significantly smaller (*p* < 0.05, *t*-test) after SHS exposure (47 ± 10 Hz vs. 16 ± 8 Hz, FA vs. SHS, respectively).

### 3.5 Potassium channel blockers on action potential characteristics

**Figure 7:**
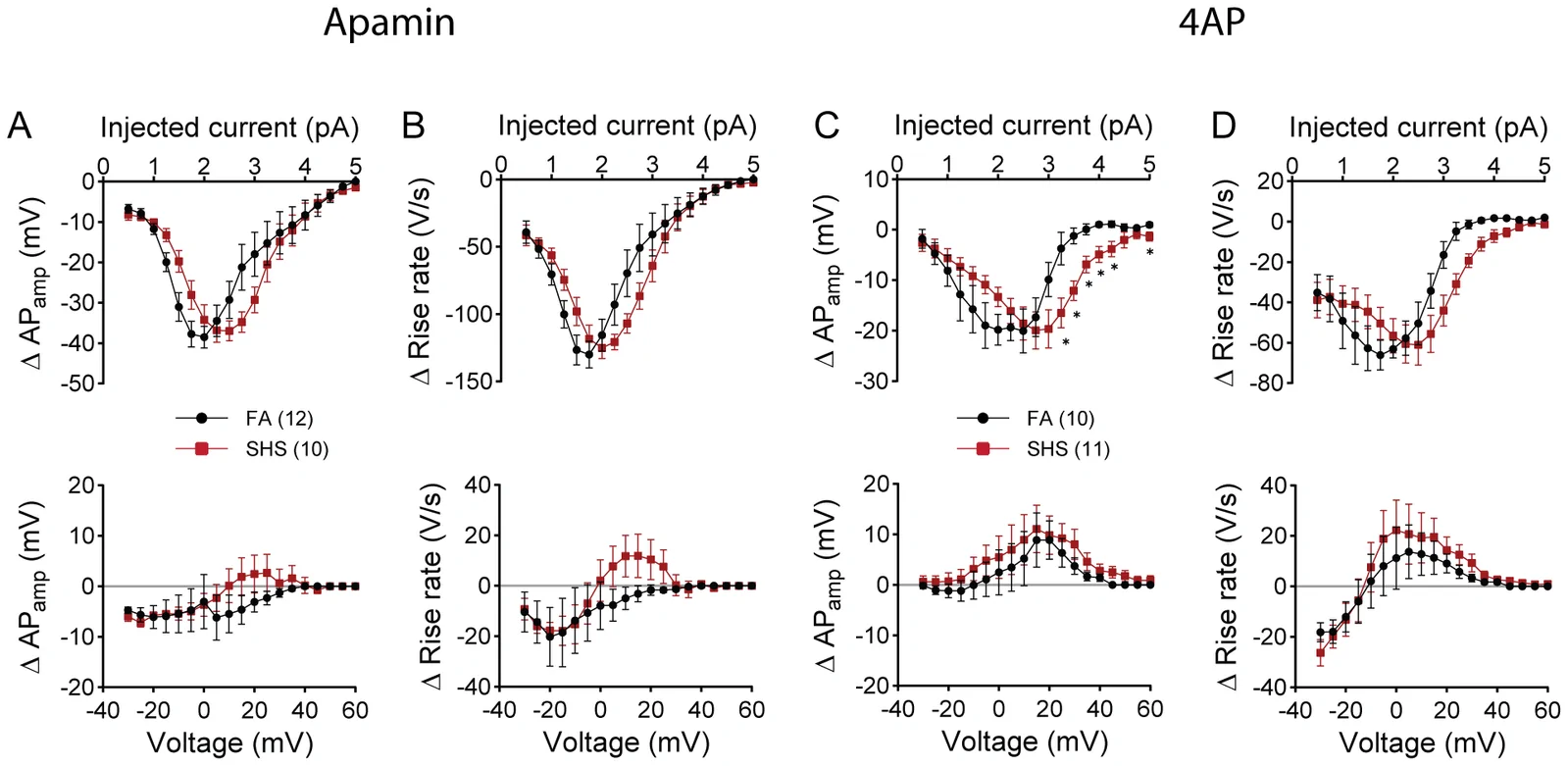
Effects of apamin and 4-aminopyridine (4AP) on action potential upstroke characteristics. **(A)** Apamin-induced changes in action potential amplitude (apamin – aCSF) plotted against injected current (top) and membrane voltage (bottom). SHS exposure did not change apamin’s effects on action potential amplitudes. **(B)** Apamin-induced decrease in action potential rise rate plotted against injected current (top) and membrane voltage (bottom). SHS exposure did not change apamin’s effects on action potential rise rate. **(C)** 4AP-induced decrease in AP amplitude plotted against injected current (top, a right-ward shift in the SHS group) and membrane voltage (bottom, no exposure effects). **(D)** 4AP-induced decrease AP rise rate at the same injected currents (top, no exposure effects) and membrane voltage (bottom, no exposure effects). Numbers in parentheses indicate number of neurons. Full mixed-effects analysis p-values are in **Supplementary Table 8**. ^*^p < 0.05 FA vs. SHS, Fisher’s LSD test.

Application of apamin significantly reduced AP amplitude at equivalent injected currents (**Figure 7A**, top). This reduction was due to a steeper apamin-induced membrane voltage response to injected currents (**Figure 6B**) because apamin had no significant effect on the AP amplitude when plotting against the membrane voltage (**Figure 7A**, bottom). Apamin also significantly reduced the AP rise rate (**Figure 7B**, top), however, SHS exposure did not impact apamin’s effect on AP maximum rise rate (**Figure 7B**). Application of 4-aminopyridine significantly reduced AP amplitude (**Figure 7C**, top) and rise rate (**Figure 7D**, top). Plotting 4-aminopyridine’s effect against membrane voltage showed that SHS exposure did not impact the effects of 4-aminopyridine on AP amplitude and rise rate (**Figures 7C, D**).

**Figure 8:**
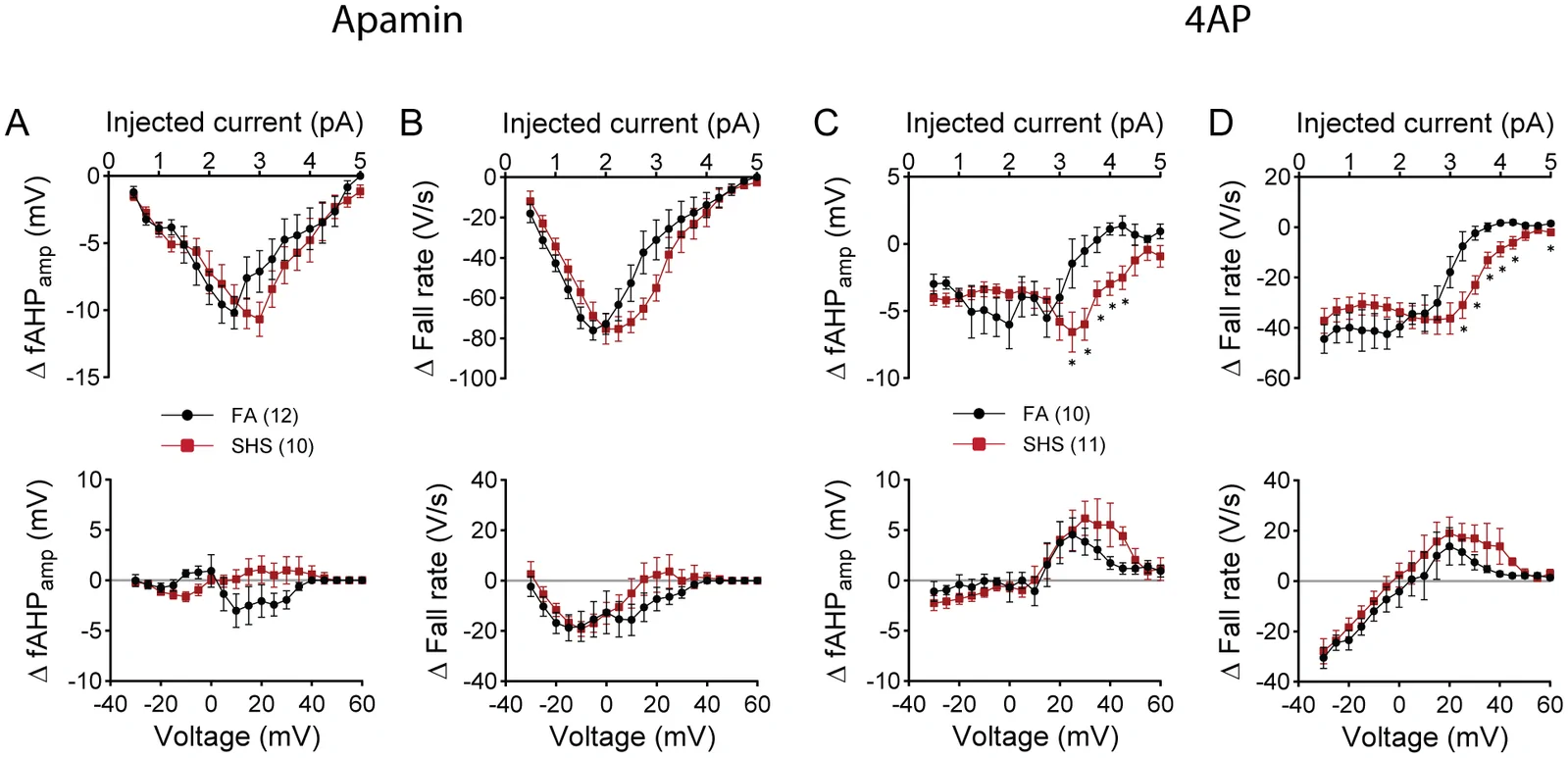
Effects of apamin and 4-aminopyridine (4AP) on action potential downstroke characteristics. **(A)** Apamin-induced changes in fAHP amplitude (4AP – aCSF) plotted against injected current (top) and membrane voltage (bottom). SHS exposure did not change apamin’s effects on fAHP amplitude. **(B)** Apamin-induced changes in action potential fall rate plotted against injected current (top) and membrane voltage (bottom). SHS exposure did not change apamin’s effects on action potential fall rate. **(C)** 4AP-induced decrease in fAHP amplitude plotted against injected current (top, a right-ward shift in the SHS group) and membrane voltage (bottom, no exposure effects). **(D)** 4AP-induced decrease AP fall rate at the same injected currents (top, a right-ward shift in the SHS group) and membrane voltage (bottom, no exposure effects). Numbers in parentheses indicate number of neurons. Full mixed-effects analysis p-values are in **Supplementary Table 9**. ^*^p < 0.05 FA vs. SHS, Fisher’s LSD test.

Apamin application decreased fAHP amplitude (**Figure 8A**, top), an effect that appears to be associated with a higher voltage response in the presence of apamin (**Figure 6B**) because expressing the apamin-induced changes as a function of membrane voltage (**Figure 8A**, bottom) did not show any significant apamin effect. Similarly, apamin decreased AP fall rate when the data were expressed as a function of injected current (**Figure 8B**, top); expressing these data as a function of membrane voltage attenuates this effect (**Figure 8B**, bottom). SHS exposure did not affect apamin’s effects on fAHP amplitude and AP fall rate. On the other hand, 4-aminopyridine significantly decreased fAHP amplitude and AP fall rate at lower injected currents (**Figures 8C, D**, top), with a right-ward shift in the SHS group. Plotting the data against membrane voltage showed no significant difference between the two exposure groups in 4-aminopyridine’s effects (**Figures 8C, D**, bottom).

## 4 Discussion

**Figure 9:**
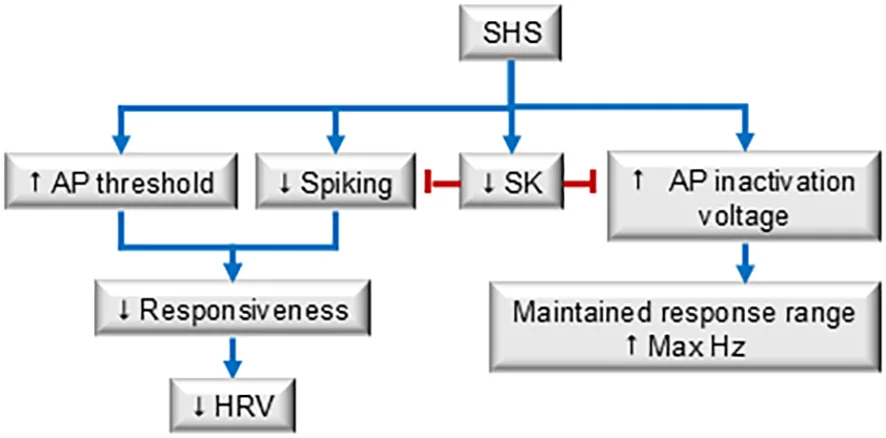
Summary of SHS’s impact on CVNs. SHS exposure decreased CVN’s excitability by increasing AP threshold and decreasing spiking response, effects that were not mediated by enhanced SK or voltage-gated potassium channel activity. The overall reduction in CVN/s responsiveness could lead to reduced heart rate variability (HRV). The reduced responsiveness was associated with decreased SK channel activity and increased AP inactivation voltage, suggesting that these adaptations could help to dampen the reduced responsiveness.

As summarized in **Figure 9**, our results showed that 12 weeks of SHS exposure significantly increased AP threshold and reduced spiking response to excitation, suggesting a reduced responsiveness of CVNs in the NA. The reduced neuronal excitability in CVNs may explain the previously reported SHS exposure-induced decreases in HRV (Pan et al., 2023). SHS exposure did not significantly alter resting membrane potential or 4-aminopyridine sensitive channel activity, suggesting that leak potassium channels and voltage gated potassium channels were unlikely to contribute to the reduced excitability. While the mechanisms underlying the reduced excitability of the CVNs remain unknown, our study revealed two adaptations that may serve to counteract the reduced excitability. First, AP inactivation at higher voltages could help to maintain the spiking response range and increase the maximum discharge frequency. Second, blocking SK channels with apamin had smaller effects on the spiking response of SHS neurons, suggesting a reduced SK channel activation during spiking response helped to dampen the reduced excitability.

It is well-recognized that activation of SK channels reduces spiking activity in neurons in central cardiovascular pathways (Chen et al., 2010; Chen and Toney, 2009; Ferreira-Neto et al., 2017; Lin et al., 2010b). For example, activation of SK channels reduces the excitability of paraventricular nucleus neurons projecting to the rostral ventrolateral medulla, while having no significant effect on the spike-frequency adaptation (Chen and Toney, 2009). Changes in SK channel function also have been shown to shape CVN’s response to excitation: Neonatal mice from diabetic mothers have lower spiking response to depolarizing current injections – a reduced excitability that has been attributed to higher apamin-sensitive currents in CVNs (Lin et al., 2010a). Additionally, application of apamin eliminated the difference in spiking response between control and diabetic dams, suggesting that augmented SK-mediated currents contribute to the reduced excitability. Our data show that blocking the SK channels with apamin resulted in a greater difference in spiking response between the FA and SHS groups. Given the role of SK channels in shaping neuronal excitability, our data suggest that the reduced SK channel activity is likely an adaptive response to counteract the SHS-induced reduction in neuronal excitability.

Many factors can directly or indirectly alter AP threshold, including sodium channel density and activation properties, steady-state inactivation of sodium channels, SK channel activity, and potassium channel properties (Hallworth et al., 2003; Platkiewicz and Brette, 2010). While both apamin and 4-aminopyridine significantly changed AP threshold (increase with apamin and decrease with 4-aminopyridine), there was no significant difference in response to these pharmacologic agents by exposure. These data suggest that the SHS exposure-induced increases in AP threshold are likely the result of mechanisms beyond SK channels and voltage gated potassium channels activity. The shift in AP inactivation to higher voltages was accompanied by unchanged maximum AP rise/fall rate, raising the possibility that a shift in activation and/or inactivation characteristics of voltage gated channels, rather than changes in channel density, may contribute to the higher AP thresholds. While characterizing sodium channel properties under voltage-clamp configuration (e.g. gating and voltage-dependence) is outside the scope of this study, it would have directly tested this possibility and strengthen the mechanistic interpretation. A thorough survey of SHS-induced changes in sodium channel properties in CVNs warrants further investigation.

The CVNs in the NA are not pacemaker cells and activation of these neurons largely rely on the excitatory glutamatergic inputs (Mendelowitz, 1996). Although SHS exposure-induced decreases in intrinsic excitability of the CVNs could contribute to the reduced HRV that follows SHS exposure, we cannot rule out the possibility that reduced synaptic excitatory inputs to the CVNs in the NA may also contribute to this phenotype. The nucleus tractus solitarius (NTS) provides direct excitatory projections to the CVNs in the NA, releasing glutamate that binds to both NMDA and non-NMDA receptors (Agarwal and Calaresu, 1992; Neff et al., 1998b). In this regard, nicotine, a unique component of SHS, has been shown to increase presynaptic glutamate release frequency and augment postsynaptic non-NMDA currents of CVNs (Neff et al., 1998a). Furthermore, inhaled SHS can evoke lung inflammatory responses and stimulate lung sensory afferents that synapse onto CNS neurons. Within the CNS, NTS is the first site that receives and integrates all sensory afferent signals, including those from the lungs. We previously showed that extended activation of the NTS through exposures to SHS, ozone, or allergens causes long-lasting changes in the excitability of these neurons (Chen et al., 2003, 2001; Sekizawa et al., 2008). Thus, it is plausible that SHS exposure-induced neuroplasticity in the NTS alters the synaptic properties in the NA, resulting in changes in CVNs excitability.

At the highest injected step current (700 pA), our results showed a 17% decrease in spiking activity after 12 weeks of SHS exposure (**Figure 2C**). This decrease is similar to what was observed after four weeks of exposure (Sun et al., 2021). Comparing effects of SHS between 4 and 12 weeks of exposure, SHS similarly increased AP threshold and right-ward shift in AP characteristics. The apamin effects were also similarly reduced in the SHS group between 4 and 12 weeks of exposure. Considering that SHS exposure decreased short-term HRV by 31% at week 4 and 18% at week 12 (Pan et al., 2023), these data raise the possibility that longer SHS exposures may cause further changes in synaptic excitabilities, since the CVN’s intrinsic excitability remains suppressed.

SHS exposure has been shown to activate macrophages to release proinflammatory cytokines leading to recruitment of inflammatory cells to the lungs, release of inflammatory mediators, and depletion of protective antioxidants in bronchoalveolar lavage fluid (Gardi and Valacchi, 2012; Li et al., 1994). Consequently, the lung epithelium becomes more permeable allowing passage of particles and diffusible mediators into the circulation (Li et al., 1994).These lung inflammatory events could change the spiking activity and patten of neurons in the autonomic pathways by 1) changing the lung sensory input to the neurons in the autonomic pathways (Bonham et al., 2001; Sekizawa et al., 2008) and/or disrupting the blood brain barrier and allowing the mediators to gain direct access to the brain neurons (Calderon-Garciduenas et al., 2002). Additionally, SHS exposure-induced lung inflammatory events and autonomic dysregulation could induce remodeling of cardiac electrical properties that further contribute to increased cardiovascular morbidity and mortality (Chen et al., 2008; Wang et al., 2018).

SK channels are widely distributed, including the central nervous system and the heart, and have been implicated in diseases. Aging and cognitive decline is associated with over-expression of SK channels. Blocking SK channels has been shown to reverse symptoms Alzheimer’s disease (Hasan et al., 2023). In the heart, heart failure is associated with increased SK channel expression and sensitivity to intracellular calcium (Chang and Chen, 2015). Pharmacological treatment of blocking SK channels in neurological disorder or heart failure may exacerbate the cardiovascular risks associated with SHS exposure. In this regard, soluble epoxide hydrolase inhibitors have been shown to inhibit SHS-induced lung inflammatory response (Wang et al., 2012) and reduced HRV (Pan et al., 2023). Whether soluble epoxide hydrolase inhibitors also reverse the effects of SHS on CVNs merits further investigation.

In conclusion, our data suggest that SHS exposure, at an environmentally relevant concentration, induces cardiac dysregulation (reduced HRV) by inducing neuroplasticity of CVNs in the NA. These findings underscore the need to protect the cardiac health of nonsmokers.

## Data Availability

The original contributions presented in the study are included in the article/**Supplementary Material**. Further inquiries can be directed to the corresponding author.
