# Supplementary material for: Extended secondhand smoke exposure alters potassium channel activity and reduces excitability of cardiac vagal neurons in mice
Source: Front Physiol. 2026 Aug 19;17:1907874. doi: 10.3389/fphys.2026.1907874 (PMC13534052; doi:10.3389/fphys.2026.1907874)
Supplement: Supplementary file 1 [file Supplementaryfile1.docx]

Supplementary Material

# 1 Supplementary Tables

Table 1: Statistical analysis p-values for Figure 2.

|  | **Exposure** | **Current** | **Interaction** |
| --- | --- | --- | --- |
| C | 0.0001 | <0.0001 | <0.0001 |
| D | 0.0013 | <0.0001 | 0.0203 |
| E | 0.7573 | <0.0001 | 0.9180 |

Panels C & D: 2-way repeated ANOVA

Panel E: Mixed-effects analysis

Table 2: Statistical analysis p-values for Figure 3.

|  | **Exposure** | **Current** | **Interaction** |
| --- | --- | --- | --- |
| F | 0.0246 | <0.0001 | <0.0001 |
| H | 0.6677 | <0.0001 | 0.6419 |
| I | 0.0947 | <0.0001 | <0.0001 |

Panels F & H: 2-way repeated ANOVA

Panel I: Mixed-effects analysis

Table 3: Statistical analysis (mixed-effects analysis) p-values for Figure 4.

|  | **Exposure** | **Current** | **Interaction** |
| --- | --- | --- | --- |
| A | 0.0124 | <0.0001 | <0.0001 |
| B | 0.0448 | <0.0001 | <0.0001 |
| D | 0.0020 | <0.0001 | <0.0001 |
| E | 0.0397 | <0.0001 | <0.0001 |

Table 4: 3-way repeated ANOVA p-values for Figure 5.

|  | **Exposure** | **Current** | **Blocker** | **Exposure**  **x**  **blocker** | **Exposure**  **x**  **Current** | **blocker**  **x**  **Current** | **3-way interaction** |
| --- | --- | --- | --- | --- | --- | --- | --- |
| A1 | 0.0112 | <0.0001 | <0.0001 | 0.0085 | 0.0162 | <0.0001 | 0.2125 |
| B1 | 0.0062 | <0.0001 | <0.0001 | 0.0210 | <0.0001 | <0.0001 | 0.0058 |
| C1 | 0.0161 | <0.0001 | 0.0163 | 0.2819 | 0.0163 | 0.0009 | 0.2900 |
| D1 | 0.0063 | <0.0001 | <0.0001 | 0.3756 | <0.0001 | <0.0001 | 0.0922 |

Table 5: 2-way repeated ANOVA p-values for Figure 5.

|  | **Exposure** | **Current** | **Interaction** |
| --- | --- | --- | --- |
| A2 | 0.0085 | <0.0001 | 0.2195 |
| B2 | 0.0210 | <0.0001 | 0.0058 |
| C2 | 0.1618 | <0.0001 | 0.1813 |
| D2 | 0.3756 | <0.0001 | 0.0922 |

Table 6: 3-way repeated ANOVA p-values for Figure 6.

|  | **Exposure** | **Current/**  **Voltage** | **Blocker** | **Exposure**  **x**  **blocker** | **Exposure**  **x**  **Current/**  **Voltage** | **blocker**  **x**  **Current/**  **Voltage** | **3-way interaction** |
| --- | --- | --- | --- | --- | --- | --- | --- |
| A1 | 0.1048 | <0.0001 | 0.0022 | 0.0242 | <0.0001 | <0.0001 | 0.0508 |
| B1 | 0.0376 | <0.0001 | <0.0001 | 0.2478 | <0.0001 | <0.0001 | 0.0039 |
| C1 | 0.0260 | <0.0001 | <0.0001 | 0.0014 | <0.0001 | <0.0001 | <0.0001 |
| D1 | 0.0821 | <0.0001 | <0.0001 | 0.5433 | <0.0001 | <0.0001 | 0.0001 |
| E1 | 0.8903 | <0.0001 | <0.0001 | 0.5750 | 0.9838 | <0.0001 | 0.9548 |
| F1 | 0.3300 | <0.0001 | 0.7409 | 0.7987 | <0.0001 | <0.0001 | 0.8189 |

Table 7: 2-way repeated ANOVA p-values for Figure 6.

|  | **Exposure** | **Current/**  **Voltage** | **Interaction** |
| --- | --- | --- | --- |
| A2 | 0.0197 | <0.0001 | 0.2350 |
| B2 | 0.2473 | <0.0001 | 0.1491 |
| C2 | 0.0077 | 0.0002 | 0.0297 |
| D2 | 0.5433 | 0.0004 | 0.0367 |
| E2 | 0.5715 | <0.0001 | 0.5545 |
| F2 | 0.1507 | <0.0001 | 0.4242 |

Table 8: 2-way repeated ANOVA p-values for Figure 7.

|  | **Exposure** | **Current/**  **Voltage** | **Interaction** |
| --- | --- | --- | --- |
| A top | 0.7314 | <0.0001 | 0.1386 |
| A bottom | 0.5775 | 0.0527 | 0.4509 |
| B top | 0.6379 | <0.0001 | 0.1215 |
| B bottom | 0.9406 | 0.0017 | 0.6978 |
| C top | 0.3426 | <0.0001 | 0.0419 |
| C bottom | 0.4697 | 0.0341 | 0.5347 |
| D top | 0.4156 | <0.0001 | 0.1342 |
| D bottom | 0.6500 | 0.0190 | 0.3390 |

Table 9: Mixed-effects analysis p-values for Figure 8.

|  | **Exposure** | **Current/**  **Voltage** | **Interaction** |
| --- | --- | --- | --- |
| A top | 0.3682 | <0.0001 | 0.5136 |
| A bottom | 0.4545 | 0.4928 | 0.0874 |
| B top | 0.5153 | <0.0001 | 0.0869 |
| B bottom | 0.5738 | 0.0010 | 0.6171 |
| C top | 0.0477 | <0.0001 | 0.0209 |
| C bottom | 0.6545 | 0.0001 | 0.4513 |
| D top | 0.4267 | <0.0001 | 0.0170 |
| D bottom | 0.5553 | <0.0001 | 0.7700 |

# 2 Supplementary Figures

**Supplementary Figure 1.** Resting membrane potential (Rem) and instantaneous frequency of the first 15 action potentials. **A.** There was no difference in resting membrane potential between the two groups (*t*-test, *p* > 0.05). **B.** Instantaneous frequency at injected currents of 500 and 600 pA showed similar spiking adaptation pattern in both groups (3-way repeated ANOVA). **C.** Instantaneous frequency of neurons discharged a total of ~20 and ~30 action potentials (APs) in response to 1s depolarizing current steps, further demonstrating that there was no difference in frequency adaptation between the two groups (3-way repeated ANOVA). **D.** *p* values of three-way repeated ANOVA results for panels B and C.

|  | **Exposure** | **Current/**  **Voltage** | **Interaction** |
| --- | --- | --- | --- |
| A | 0.0023 | <0.0001 | <0.0001 |
| B | 0.0024 | <0.0001 | <0.0001 |
| C | 0.0013 | <0.0001 | <0.0001 |
| D | 0.4752 | <0.0001 | 0.0192 |
| E | 0.0270 | <0.0001 | 0.0001 |
| F | 0.0018 | <0.0001 | 0.0004 |

G

**Supplementary Figure 2.** Action potential (AP) amplitude (**A**) and rise rate (**B**) curves shifted to the right in the SHS-exposed group. fAHP amplitude (**C**) and AP fall rate (**D**) also shifted to the right in the SHS-exposed group. CVNs from the SHS group had narrower ½ width at higher injected currents (**E**) and voltages (**F**). **G.** Mixed-effects analysis p-values. * *p* < 0.05 FA vs. SHS, Fisher’s LSD test.
